# B355252 Suppresses LPS-Induced Neuroinflammation in the Mouse Brain

**DOI:** 10.3390/brainsci14050467

**Published:** 2024-05-07

**Authors:** Qingping He, Qi Qi, Gordon C. Ibeanu, P. Andy Li

**Affiliations:** 1 Department of Pharmaceutical Sciences, Biomanufacturing Research Institute and Technology Enterprise (BRITE), College of Health and Sciences, North Carolina Central University, Durham, NC 27707, USA; qhe@nccu.edu (Q.H.); gibeanu@nccu.edu (G.C.I.); 2 Human Vaccine Institute, Department of Surgery, Duke University Medical Center, Durham, NC 27707, USA; qi.qi@duke.edu

**Keywords:** astrocyte, B355252, brain, cytokine, lipopolysaccharide, microglia, neuroinflammation, pyroptosis, NLRP3

## Abstract

B355252 is a small molecular compound known for potentiating neural growth factor and protecting against neuronal cell death induced by glutamate in vitro and cerebral ischemia in vivo. However, its other biological functions remain unclear. This study aims to investigate whether B355252 suppresses neuroinflammatory responses and cell death in the brain. C57BL/6j mice were intraperitoneally injected with a single dosage of lipopolysaccharide (LPS, 1 mg/kg) to induce inflammation. B355252 (1 mg/kg) intervention was started two days prior to the LPS injection. The animal behavioral changes were assessed pre- and post-LPS injections. The animal brains were harvested at 4 and 24 h post-LPS injection, and histological, biochemical, and cytokine array outcomes were examined. Results showed that B355252 improved LPS-induced behavioral deterioration, mitigated brain tissue damage, and suppressed the activation of microglial and astrocytes. Furthermore, B355252 reduced the protein levels of key pyroptotic markers TLR4, NLRP3, and caspase-1 and inhibited the LPS-induced increases in IL-1β, IL-18, and cytokines. In conclusion, B355252 demonstrates a potent anti-neuroinflammatory effect in vivo, suggesting that its potential therapeutic value warrants further investigation.

## 1. Introduction

B355252[4-chloro-N-(naphthalen-1-ylmethyl)-5-(3-(piperazin-1-yl)phenoxy)thiophene-2-sulfonamide], a compound synthesized in 2010 by Dr. Williams at North Carolina Central University, shows promise in promoting neurite growth and differentiating neuron-like NS-1 cells in vitro [1,2]. Later, it was demonstrated that B355252 protects murine hippocampal neuronal HT-22 cells against glutamate-mediated cytotoxicity, 6-hydroxydopamine-induced cell death, and cobalt chloride-induced chemical hypoxic damage [3,4,5]. To date, the known mechanisms of action include inhibiting calcium influx into the cell, reducing reactive oxygen species (ROS) formation, stabilizing mitochondrial membrane potential, decreasing apoptosis inducing factor (AIF) nuclear translocation, suppressing BAX and caspase-3 activation, limiting autophagy induction, and activating ERK3 signaling pathway [3,4,5,6,7]. B355252 has been assessed in only one in vivo study using cerebral ischemic model [8]. In this study, B355252 demonstrated a neuroprotective effect against endothelin-1-cerebral-injection-induced focal ischemia. Since B355252 is a small phenoxy thiophene sulfonamide molecule and its analog B355227 has been shown to be capable of passing through the blood–brain barrier (BBB) [9], additional in vivo studies are needed to explore its therapeutic effects in various animal disease models prior to its potential clinical translation.

Neuroinflammation, an innate immune response of the central nervous system (CNS), can lead to neuronal damage in pathological conditions [10]. It is implicated in infectious disorders, autoimmune diseases, strokes, and neurodegenerative diseases like Parkinson’s and Alzheimer’s [11,12,13]. Pyroptosis, an inflammation-induced form of cell death, involves the activation of inflammasomes and caspase-1, leading to the release of pro-inflammatory factors and cell membrane rupture. Lipopolysaccharides (LPS) injection to the rodents has been established as an in vivo inflammatory animal model [14]. In the brain, it induces activations of microglia and astrocyte and productions of various inflammatory cytokines, which could result in pyroptotic type of cell death [15].

Pyroptosis is initiated when inflammatory cytokines bind to receptors on the cell membrane or cytoplasm, triggering the formation of an inflammasome comprising Nod-like receptors protein-3 (NLRP3), apoptosis-related speckle protein (ASC), and procaspase-1 [16,17]. Activation of caspase-1 by the inflammasome leads to the secretion of pro-inflammatory factors IL-1β and IL-18, as well as the release of the N-terminal fragment of Gasdermin D (GSDMD). The N-terminal fragment of GSDMD forms pores in the cell membrane, causing cell swelling, osmosis, and ultimately cell death, known as pyroptosis [18,19,20,21]. Pyroptosis is a programmed cell death type characterized by early plasma membrane rupture, releasing intracellular components that exacerbate inflammation [22,23]. This study aims to explore the potential anti-inflammatory effects of B3553252 in mice injected with lipopolysaccharides (LPS), which is known to induce neuroinflammation.

## 2. Materials and Methods

### 2.1. Reagents and Antibodies

LPS from *E. coli* 0111: B4 was purchased from Invitrogen (Waltham, MA, USA). A stock solution was made in 5 mg/mL in distilled water and freshly diluted to 1 mg/mL with 0.9% saline prior to each injection. B355252 (BML1007-10 mg, Sigma-Aldrich, St. Louis, MO, USA) was dissolved in 2% DMSO in 1 mg/kg for animal intraperitoneal injections.

The following rabbit or mouse antibodies were purchased from Cell Signaling Technology (Denvers, MA, USA): Iba1 (cat #17198s), GFAP (cat #12389), Cleaved Caspase-1 (Asp296, 2G2I, cat #89332S), NLRP3 (D4D8T, cat #15101S), Toll-like Receptor 4 (D8L5W, cat #14358S), IL-1β (D3H1Z, cat #12507S), and IL-18 from Invitrogen (cat #PA5-76082).

### 2.2. Animal Cohorts Assignment and Behavior Assessment

Specific pathogen-free male and female adult C57BL/6J mice aged 3–7 months old and weighing 20–35 g were obtained from Jackson Laboratories. All in vivo experiments adhered to the NIH Guide for the Care and Use of Laboratory Animals and were approved by the Institutional Animal Care and Use Committee (IACUC) at North Carolina Central University (NCCU). The mice were housed in plastic cages under controlled conditions (12/12 light–dark cycle, temperature of 22 ± 2 °C, and humidity at 50 ± 10%, with free access to water and standard food).

**Figure 1:**
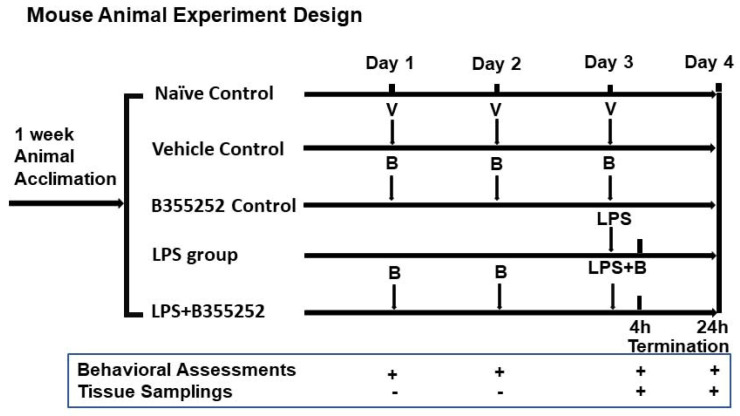
Schematic diagram of animal experimental design. V, vehicle; B, B355252.

The animals were randomly allocated to the following seven groups, as presented in Figure 1: (1) naïve control (*n* = 10), receiving no drug treatment; (2) vehicle control (*n* = 7), administered 2% DMSO; (3) B355252 control (*n* = 5), treated with B355252 (1 mg/kg) for 3 days only; (4) LPS4h group (*n* = 6), euthanized 4 h after LPS injection; (5) LPS24h group (*n* = 11), euthanized 24 h after LPS injection; (6) LPS4h + B group (*n* = 4), pretreated with B355252 for 2 days, followed by simultaneous injections of LPS plus B355252 on the third day and euthanized after 4 h; and (7) LPS24h + B group (*n* = 9), pretreated with B355252 for 2 days, followed by simultaneous injections of LPS plus B355252 on the third day and euthanized after 24 h. All animals were included in the behavioral assessments. Four animals in each group were used for morphological studies and biochemical analysis. LPS was dissolved in distilled water and B355252 in 2% DMSO. Daily intraperitoneal (I.P.) injections of B355252 (1 mg/kg) commenced 2 days before the single-LPS-dose injection (1 mg/kg).

Animal appearance and behavior were evaluated using a method outlined by Paster et al. (2009), including assessments of appearance, natural behavior, provoked behavior, and body condition, with scores ranging from 1 to 3 recorded daily before and after B355252 and/or LPS treatments [24].

### 2.3. Tissue Sampling

At the designated endpoints, the mice were euthanized via CO_2_ inhalation. Their brains were then harvested and dissected on ice. One hemisphere was promptly frozen in liquid nitrogen for future biochemical analysis, while the other hemisphere was fixed in 4% formaldehyde solutions for histological and immunohistochemical evaluations. Additionally, a separate set of fresh brain samples were embedded into optimal cutting temperature (OCT) compound medium, immediately frozen on dry ice, and then stored at −80 °C.

### 2.4. Histopathology and Hematoxylin/Eosin (H&E) Staining

Following 24 h of fixation in 4% formaldehyde, the brain tissues were processed using a Leica Tissue Processor according to a standard protocol for mouse tissues. In summary, the tissues were dehydrated in a gradient of ethanol (70%, 95% and 100%), cleared in xylene, infiltrated with paraffin wax, and embedded into paraffin blocks. These blocks were then sliced at a thickness of 5 µm using a microtome (Leica, Wetzlar, Germany). The sections were stained with H&E and examined using an Axio Observer Inverted Microscopy (Zeiss, Munich, Germany).

For each section, four microscopic fields at 40× magnification were captured in the cortex, caudoputamen (Cpu), and hippocampus and subsequently quantified. Cells exhibiting bright red eosinophilic staining with condensed triangular nuclei were identified and counted as damaged neurons.

### 2.5. Immunohistochemistry (IHC) and Immunofluorescence (IF)

Tissue samples for IHC were prepared from paraffin-embedded sections at a thickness of 5 μm on positively charged frosted glass slides. Immunostainings were conducted using the indirect peroxidase-labeling method provided in the VECTASTAIN ABC-HRP kit (Vector Laborotaries, Newark, CA, USA). Initially, sections were dewaxed and underwent heat-induced antigen retrieval in 10 mM sodium citric (pH 6.0) within a pressure cooker for 15 min. Endogenous peroxidase activity was blocked using 3% H_2_O_2_ in methanol for 30 min, followed by incubation with diluted normal blocking serum for 45–60 min, and subsequently incubation with the respective primary antibodies overnight at 4 °C. The tissue sections were then treated with appropriate biotin-conjugated secondary antibodies and developed using avidin-conjugated horseradish peroxidase (HRP) with diaminobenzidine (DAB) as a substrate. Mayer’s hematoxylin was used for counterstaining, and the slides were mounted with Permount mounting medium. Imaging and quantification of the slides were performed using deconvolution of the IHC image via NIH Image J Fiji software (version 1.51) following a published protocol [25,26].

For IF labeling, fresh mouse brain tissues in OCT embedded blocks were utilized, and the cryo-sections were prepared by cutting OCT blocks at 10 µm using a cryostat (Leica). The frozen sections were fixed in 4% paraformaldehyde for 30 min, permeabilized with 1% BSA serum in 0.4% Triton X-100 in PBS for 15–20 min, blocked with 5% BSA in 0.1% Triton x-100 in PBS for 45 min at room temperature, and then incubated with primary antibodies at appropriate dilutions in 1% BSA in PBS overnight at 4 °C. Secondary antibodies conjugated either with Alexa-488 goat anti-rabbit IgG or Alexa-647 goat anti-rabbit diluted with 1% BSA in PBS were applied and incubated for 1 h at room temperature. The sections were mounted with an anti-fade mounting medium containing DAPI or propidium iodide for nuclei labeling (Vectashield, Newark, CA, USA). Imaging of the labeled tissue slides was conducted using a Zeiss LSM 800 Laser Confocal Microscope. The captured images were quantified for mean fluorescence intensity from four fixed microscopic fields per image using NIH Image J Win32 software.

### 2.6. Nuclear and Cytosolic Fractionation

Nuclear and cytosolic fractionation procedures were conducted using the BeadBug-6 Microtube Homogenizer (SKUD1036, Benchmark Scientific Inc., Sayreville, NJ, USA) for processing mouse brain tissue lysates. Frozen brain tissues were dissected on ice, weighted, and placed in a 2.0 mL tube prefilled with 3.0 mm Zirconium beads. Subsequently, 600 μL of homemade cytosol fractionation buffer containing 15 mM Tris Base/HCl, pH 7.7, 0.25 M sucrose, 15 mM NaCl, 1.5 mM MgCl_2_, 2.5 mM EDTA, 0.25 mM Na_3_VO_4_, 25 mM NaF, 1 mM EGTA, 2 mM NaPPi, 1 mM DTT, 5 ug/mL leupeptin, 1 ug/mL Pepstatin A, 2.5 ug/mL aprotinin, 0.1% NP-40, and 0.5 mM PMSF with protease inhibitors cocktail was added. The tissue homogenization process followed a protocol recommended by the manufacturer. Briefly, the program was set up for 3 cycles at 4300 rpm speed, 30 s processing time, and 5 min rest on ice, periodically vortexing until homogenized thoroughly.

Upon completion of the homogenization processes, the lysates were collected and centrifuged at 900× *g* for 10 min at 4 °C, resulting in supernatant S1 and pellet P1 fractions. The S1 was underwent further centrifugation at 20,000× *g* for 20 min, and the resulting supernatant was designated as the cytosolic fraction. The pellet P1 fraction was washed with PBS buffer, resuspended in 300 μL RIPA lysis buffer containing 1% SDS with protease/phosphatase inhibitors cocktail, sonicated on ice (setting time 10 s, 20% Amplitude, 3 cycles), then centrifuged at 20,800× *g* for 30 min at 4 °C, the resulting supernatants were identified as the nuclear fraction. Both the cytosolic and nuclear fractions were stored at −80 °C till further analyses. Protein concentrations were determined using the Pierce BCA protein assay kit (Thermo Fisher Scientific, Waltham, MA, USA. cat# 23235).

### 2.7. Western Blot Analysis

Western blot analysis was conducted by loading an equal amount (30 μg protein) of homogenized brain tissue samples from each group loaded into individual lanes of a 4–12% Bis–Tris gel (Invitrogen). Following electrophoresis, the proteins were transferred to an immobilon–PVDF membrane (Millipore, Billerica, MA, USA). The membranes were then incubated with primary antibodies against IL18 (1:1000) and Beta actin (1:5000). After overnight incubation with primary antibodies at +4 °C, the membranes were exposed to secondary antibodies, including IDye800 donkey anti-rabbit IgG, IDye800 goat anti-mouse IgG, or IDy680 donkey anti-rabbit IgG (Li-Cor Biotechnology, Lincoln, NE, USA). Subsequently, the membranes were scanned using Odyssey CLX (Li-Cor) and analyzed with Image Studio version 5.2 for image acquisition and quantification.

### 2.8. Cytokine Array

For mouse brain homogenates from experimental normal control (NC) and LPS24h groups, LPS24h + B355252 1 mg/kg and B355252 1 mg/kg alone were used. The brain samples (*n* = 4 in each group) were pooled, homogenized, and lysed in lysis buffer provided with a cytokine array kit (ab133993, Abcam, Cambridge, UK) mixed with 1% protease and phosphatase inhibitor cocktail (Thermo Fisher Scientific™, Waltham, MA, USA. cat# 78440). Protein concentration was measured with a Pierce BCA protein kit (Thermo Scientific, cat# 23225). The cytokine array experiment was performed using a Proteome Profiler Mouse XL Cytokine Array (cat# ARY028, R&D Systems, Minneapolis, MN, USA) according to the manufacturer’s instructions. Briefly, antibody-spotted membranes were treated with a blocking solution and then incubated with 250 µg of total protein from mouse brain tissue lysate from each group overnight at 4 °C. The following day, the membranes were washed to remove unbound material followed by incubation with a cocktail of biotinylated detection antibodies. Streptavidin–HRP and chemiluminescent detection reagents were then applied, and signals were produced at each capture spot corresponding to the amount of protein bound were viewed on an iBright FL1500 Imaging System (Thermo Fisher Scientific). Data analysis was performed using the NIH ImageJ program (Win32) with the Protein Array Analyzer plug in [27].

### 2.9. Statistical Analysis

The data were presented as mean ± SD. Parametric data were analyzed using one-way ANOVA followed by the post Scheffé test. Non-parametric data were assessed using the Kruskal–Wallis test followed by the Mann–Whitney *U*-test, which are robust with smaller sample sizes. Statistical significance was defined as *p* < 0.05. Symbols denoting significance levels were used as follows: * *p* < 0.05, ** *p* < 0.01, versus control; # *p* = 0.05, ## *p* = 0.01, versus LPS counterparts.

## 3. Results

### 3.1. B355252 Improved Animal Behavior Altered by LPS

**Figure 2:**
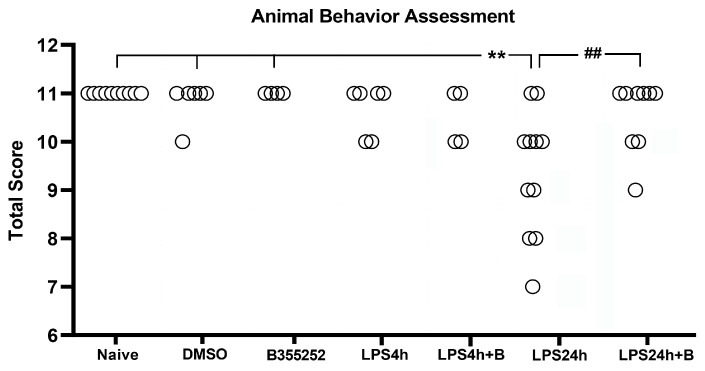
Total animals behavioral score in experimental groups. LPS impaired behavioral performance at 24 h post-LPS injection, and B355252 improved the behavioral score. ** for *p* < 0.01 vs. naïve, DMSO, and N355252 controls; ## for *p* < 0.01 vs. LPS24h.

Fifty-two animals were divided into the following groups: naïve control (*n* = 10), DMSO control (*n* = 7); B355252 control (*n* = 5); LPS4h (*n* = 6); LPS4h + B (*n* = 4); LPS24h (*n* = 11); and LPS24h + B (*n* = 9) groups. Animal behavioral scores, including the assessments of appearance, natural behavior, provoked behavior, and body condition, were recorded starting from day 3 prior to LPS injection. Prior to LPS injection, animals in all groups had a total normal score of 11 (Figure 2), and neither DMSO nor B355252 injection affected the animal behavioral scores at any time.

After 4 h of LPS injection (LPS4h group), two animals scored 10, and B355252 did not improve the animal behavioral scores. Following 24 h of LPS injection (LPS24h group), there was an increase in the number of animals exhibiting behavioral deficit, with 9 out of 11 animals scoring lower than the expected score of 11 (*p* < 0.01 vs. naïve, DMSO, and B355252 control groups). Among these nine animals, four scored 10, two scored 9, two scored 8, and one scored 7 in the LPS 24 groups.

B355252 treatment significantly improved the behavioral score compared with LPS-injected animals at 24 h, with six animals scoring 11, two scoring 10, and one scoring 9 (*p* < 0.01 LPS24h + B vs. LPS24h). Further analysis revealed that LPS primarily inhibited the animal natural behavior (reduced active body movement), and to a lesser extent, it provoked behavior change (slow reaction to stimulus), followed by changes to appearance and body condition. B355252 treatment improved animal natural behavior and completely abolished the LPS-induced behavior change. 

### 3.2. B355252 Ameliorated LPS-Induced Brain Tissue Damage

Brain sections at Bregma 0.02 and −1.94 mm levels were subjected to H&E staining. Dead neurons were identified as eosinophilic cells with shrunken and darkly stained nuclei surrounded by a void space. In the cortex and the Cpu of control animals, neurons exhibited a round shape and nuclei were clearly visible.

**Figure 3:**
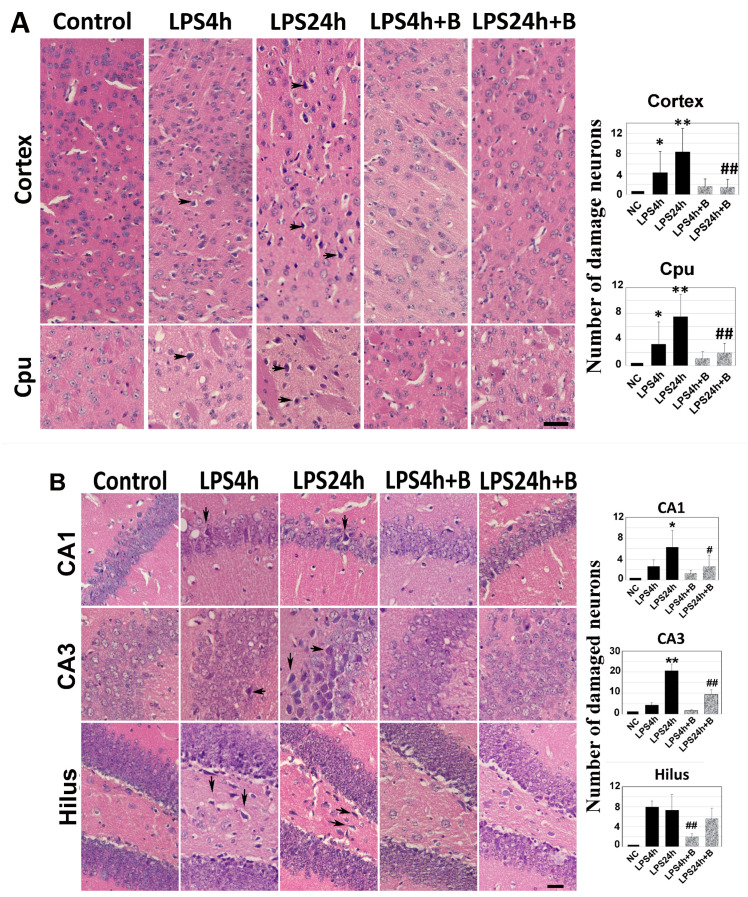
Histologic outcomes in LPS and B355252 treated animals: (**A**) H&E staining in the cortex and caudate putamen (Cpu); (**B**) H&E staining in the hippocampal CA1, CA3, and hilus. LPS administration led to increased cell death at 24 h in the cortex, Cpu, CA1, CA3, and hilus, while treatment with B355252 ameliorated the damage. The data presented in the bar graphs represent the mean +/− SD. Significance levels are denoted as *, ** for *p* < 0.05, 0.01 vs. NC (naïve control); and #, ## for *p* < 0.05, 0.01 vs. the respective LPS counterpart. The scale bar represents 100 µm.

At 4 h post-LPS injection, noticeable neuronal damage was observed in both the cortex and Cpu (arrows in Figure 3A). By 24 h post-LPS injection, the number of dead neurons significantly increased. Treatment with B355252 protected the neurons in both the cortex and the Cpu from LPS-induced damage at 24 h time point (Figure 3A).

Similarly, in the hippocampal CA1, CA3, and hilus regions (Figure 3B), virtually no damaged neurons were observed in control animals. However, in the LPS4h group, damaged neurons began to appear in the CA1, CA3, and hilus subregions. Compared with other regions, the number of damaged neurons in the hilus region was significantly high. The number of dead neurons further increased in the LPS24h group (arrows in Figure 3B). Pretreatment with B355252 significantly reduced the number of dead neurons in the CA1 and CA3 sub-regions and moderately decreased neuronal damage in the hilus area at both the 4 h and 24 h time points.

Comparison of TUNEL staining between LPS- and B355252-treated animals at 24 h further confirmed the findings observed from H&E staining. Thus, virtually no TUNEL-positive cells were observed in the control group, and LPS caused a surge of TUNEL positively stained cells (labeled in green) in the cortex, Cpu, CA1, CA3, and hilus. B355252 significantly reduced the numbers of TUNEL-positive cells in all five observed regions, with a more pronounced effect observed in the cortex and CA1 than in the other regions (Supplemental Figure S1).

### 3.3. B355252 Suppressed the Activation of Microglial and Astrocyte Cells Elicited by LPS

**Figure 4:**
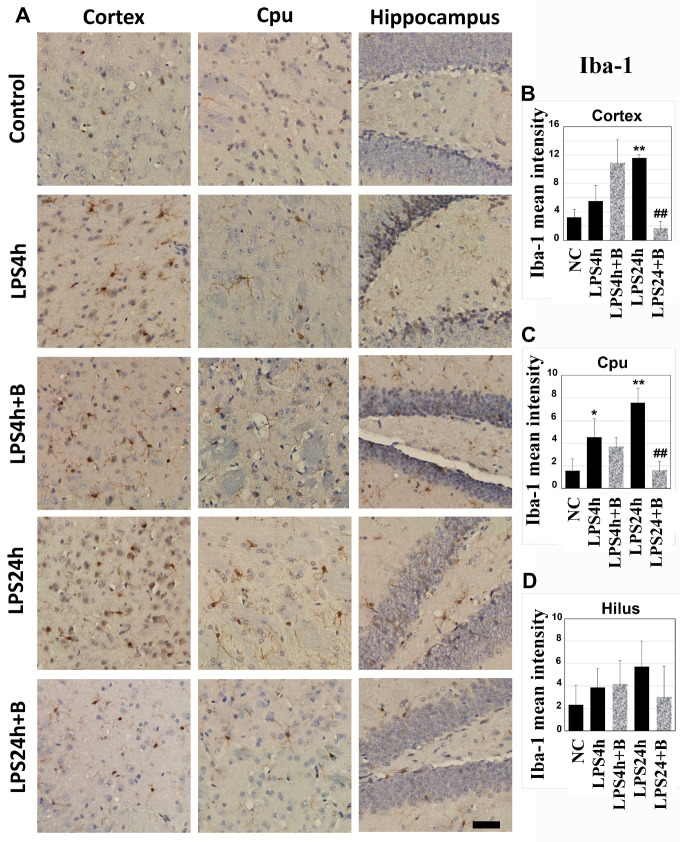
Immunohistochemistry of microglial cells labeled by Iba-1 in the cortex, Cpu, and hilus: (**A**) representative photomicrographs; (**B**–**D**) summarized mean Iba-1-positive staining intensity. LPS significantly increased the number of microglial cells after 24 h in the cortex and Cpu, while B355252 successfully decreased the numbers of Iba-1-labeled microglial cells in the cortex and Cpu. Data in bar graphs are presented as mean +/− SD. Significance levels are denoted as *, ** for *p* < 0.05, 0.01 vs. NC (naïve control); ## for *p* < 0.01 vs. LPS24h. The scale bar represents 100 µm.

Microglial activation serves as a critical indication of brain inflammation. Iba-1 immunohistochemistry was utilized to detect microglial cells on brain sections from the cortex, Cpu, and hippocampal hilus areas (Figure 4). In the control animals, microglial cells appeared as densely stained brown cells with minimal dendrites extensions. Following 4 h of LPS injection, the number of Iba-1stained cells moderately increased in the cortex and Cpu, accompanied by enlarged cell bodies and a higher number of dendrites. This increase was amplified after 24 h of LPS injection in the cortex and Cpu (*p* < 0.01 vs. NC), indicating microglial activation.

Although pretreatment of B355252 did not reduce the number of microglial cells and dendrites morphology after 4 h of LPS injection, it significantly suppressed the microglial activation in the cortex and Cpu after 24 h of LPS injection (*p* < 0.01, LPS24h + B vs. LPS24h).

In the hippocampal hilus region, LPS induced a mild-to-moderate increases in Iba-1 positively stained microglial cells after 4 and 24 h; however, these increases did not reach statistical significance due to variations. Similarly, though B355252 reduced the number of Iba-1-positive cells to 50% of the LPS24h group in the hilus, this difference did not achieve statistical significance due to variations within both the LPS24h and LPS24h + B groups (Figure 4).

**Figure 5:**
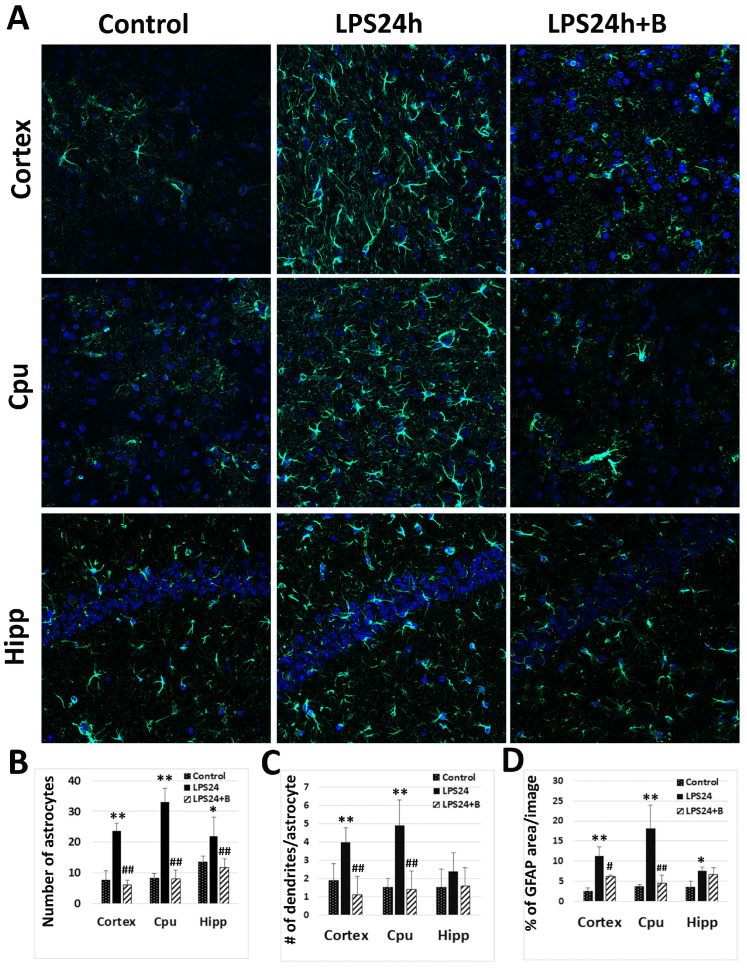
Effect of B355252 on astrocyte activation detected by GFAP immunofluorescent labeling: (**A**) representative images of GFAP labeling (GFAP-positive astrocytes are labeled in green and nuclei in blue by DAPI); (**B**) number of astrocytes in each experimental group; (**C**) mean number of dendrites per cell; (**D**) mean percentage of GFAP positively labeled area in each group. LPS markedly increased the number of astrocytes, the number of dendrites per astrocyte, and the percentage of GFAP positively labeled area. B355252 profoundly reduced these counts in the cortex and Cpu. Data in the bar graphs are presented as mean +/− SD. Significance levels are denoted as *, ** *p* < 0.05, 0.01 vs. NC (naïve control); #, ## for *p* < 0.05, 0.01 vs. LPS24h.

Astrocyte activation represents another significant response of brain tissue to inflammatory insults. Following LPS injection, there was a notable increase in the number of GFAP positively labeled astrocytes, the number of dendrites per cell, and the percentage area of GFAP staining after 24 h, indicating astrocytes activation. However, treatment with B355252 resulted in a significant reduction in the number of astrocytes, number of dendrites, and the area of GFAP staining (Figure 5).

### 3.4. B355252 Reduced Protein Levels of TLR4

**Figure 6:**
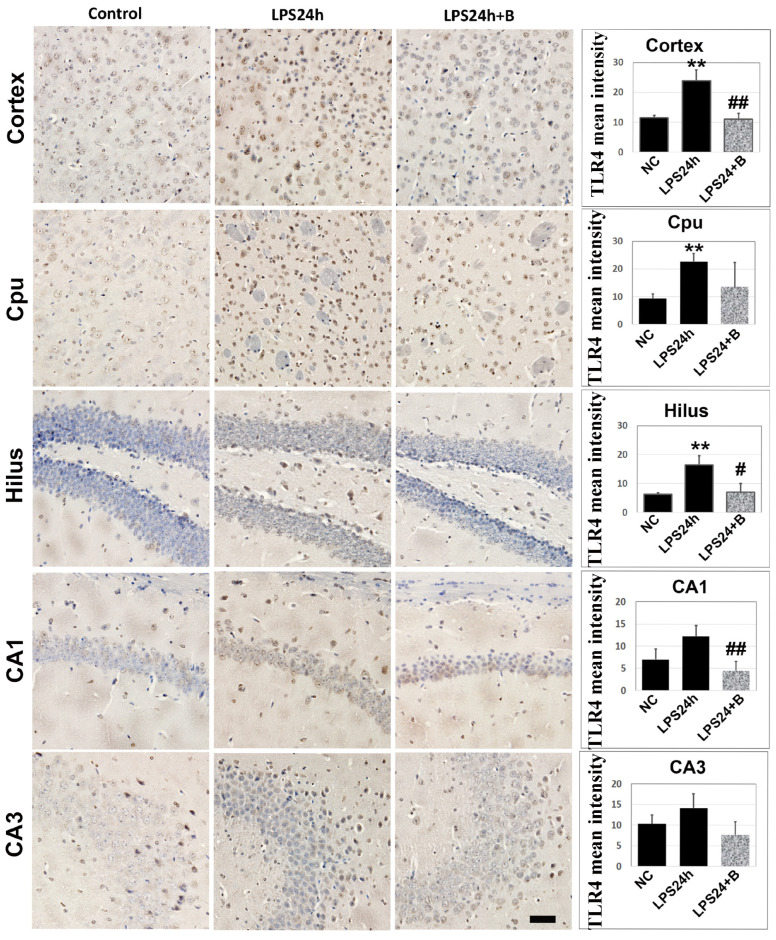
Immunohistochemistry of TLR4 in LPS- and B355252-treated animals. LPS significantly increased the TLR4 immunoreactivity in the cortex, Cpu, and hilus, while B355252 prevented these increases in the cortex and hilus. Data in the bar graphs are mean +/− SD. Significance levels are denoted as ** *p* < 0.01 vs. NC (naïve control); #, ## for *p* < 0.05, 0.01 vs. LPS24h. Bar = 100 μm.

LPS is known to activate TLR4 [28]; however, it is not known as to whether B355252 can suppress LPS-induced TLR4 elevation. The anti-TLR4 antibody was utilized to assess TLR4 immunoreactivity in brain sections. The results revealed a significant increase in the mean intensity of brown precipitates, indicating TLR4-positive immunostaining in the cortex, Cpu, and hippocampal hilus at 24 h post-LPS injection (*p* < 0.01 vs. NC; Figure 6A). While LPS slightly enhanced TLR4 reactivity in the hippocampal CA1 and CA3 regions, this enhancement did not reach statistical significance.

Treatment with B355252 treatment led to a decrease in the mean TLR4 staining intensity in the cortex, hilus, and CA1 areas (*p* < 0.01 LPS24h + B vs. LPS24h). Additionally, B355252 reduced TLR4 immunoreactivity in the Cpu and CA3; however, due to large variation, these reductions did not reach statistical significance.

### 3.5. B355252 Lowered the Levels of NLRP3 and Caspase-1

**Figure 7:**
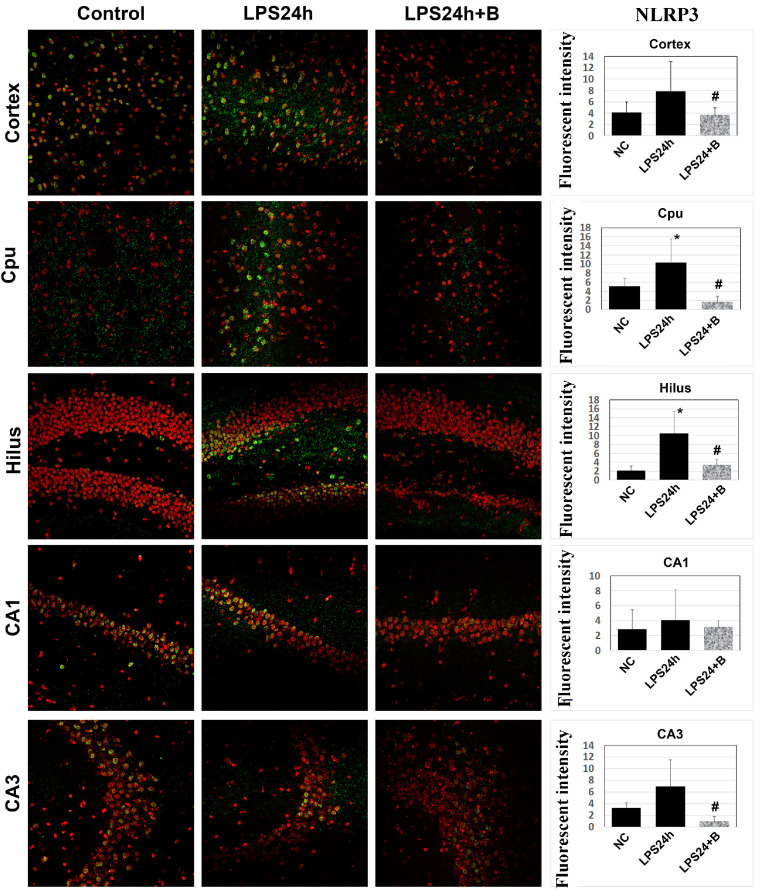
Effect of B355252 on NLRP3 in LPS-injected animals. NLRP3-positive cells are labeled in green, and nuclei are stained in red with propidium iodide. Following 24 h of LPS injection, there was an increase in the NLRP3 immunoreactivity in the Cpu and hilus. Conversely, B355252 reduced the NLRP3 fluorescent intensity in the cortex, Cpu, hilus, and CA3 regions. Data in the bar graphs are presented as mean +/− SD. * for *p* < 0.05 vs. NC; # for *p* < 0.05 vs. LPS24h.

TLR4 is known to activate the NLRP3 inflammasome pathway [29,30], which leads to caspase-1 activation by cleavage. Immunofluorescent labeling of NLRP3 showed that LPS resulted in a significant increases in the mean NLRP3 fluorescence intensity in the cortex, Cpu, and hilus (Figure 7). Treatment with B355252 decreased NLRP3 immunoreactivity in these three regions, as well as in the CA3. However, in the CA1 sub-region, there were no differences in NLRP3 levels among the three experimental groups (Figure 7).

**Figure 8:**
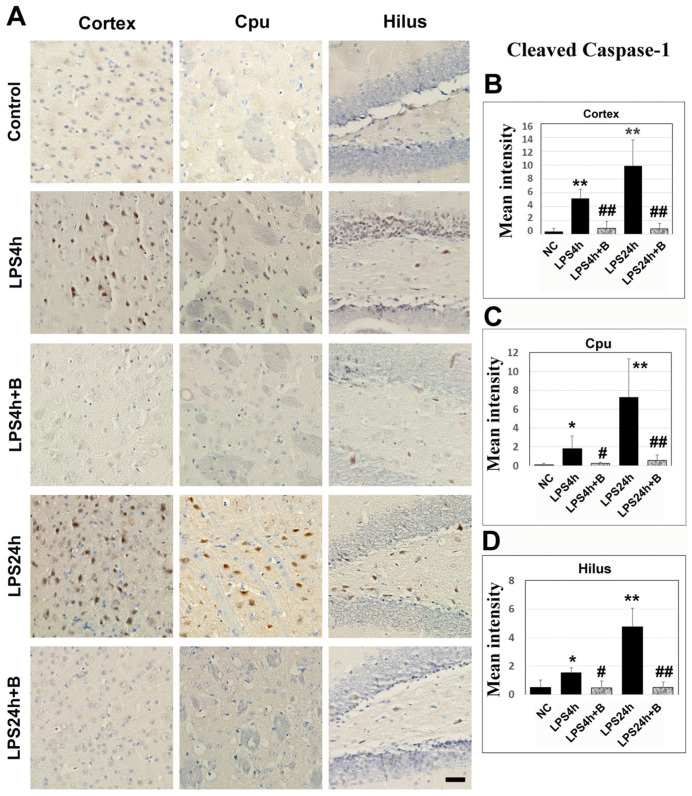
Caspase-1 immunohistochemistry in B355252-treated and LPS-injected animals. (**A**) Representative photomicrographs of caspase-1 staining. Positive cells are labeled in brown, and nuclei are counterstained with hematoxylin. The scale bar = 100 µm. (**B**–**D**) Summarized caspase-1 mean intensity in the cortex, Cpu, and hilus. LPS increased the caspase-1imuunoreactivity after 4 h and 24 h in the cortex, Cpu, and hilus. However, B355252 prevented these elevations in all three areas at both time points. Data are presented as mean +/− SD. Significance levels are denoted as *, ** for *p* < 0.05 and *p* < 0.01 vs. NC; #, ## for *p* < 0.05, 0.01 vs. LPS24h.

Immunohistochemistry of cleaved caspase-1 revealed that LPS led to moderate increases in caspase-1 positively stained cells in the cortex, Cpu, and hilus after 4 h compared to the control group. Following 24 h of LPS injection, there was a marked further increase in the numbers of caspase-1-positive cells in these three structures. Treatment with B355252 significantly reduced the number of caspase-1-positive cells both at 4 h and 24 h post-LPS injection (Figure 8).

### 3.6. B355252 Reduced the Levels of IL-1β and IL-18

**Figure 9:**
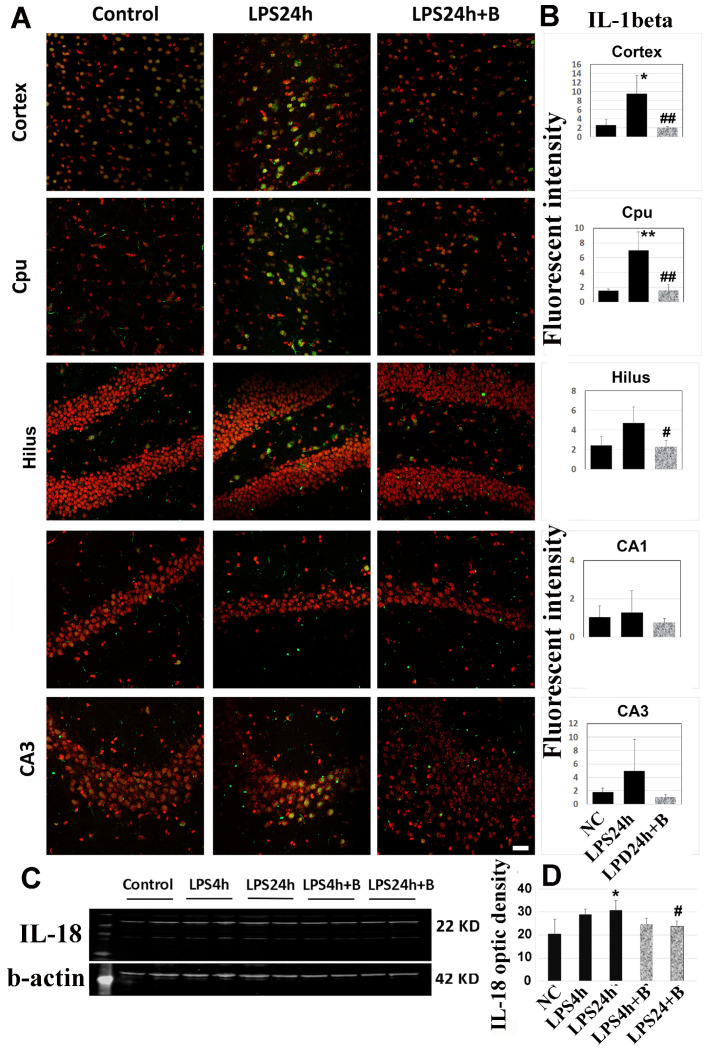
Effects of B355252 on IL-1β (**A**,**B**) and IL-18 (**C**,**D**). LPS increased the immunoreactivity of IL-1β in the cortex and Cpu, while B355252 suppressed the increases. LPS also increased the protein level of IL-18 in the cortex at 24 h post-LPS injection, and B355252 reduced this elevation. Data are presented as mean +/− SD. *, ** *p* < 0.05 and *p* < 0.01 vs. NC; #, ## *p* < 0.05, 0.01 vs. LPS24h.

Activated caspase cleaves IL-1β and IL18, resulting in enhanced inflammatory responses. As depicted in Figure 9A,B, immunohistochemistry of IL-1β revealed a significant increase in IL-1β immunoreactivity after 24h of LPS injection in the cortex and Cpu (*p* < 0.01 vs. control). The IL-1β level was also elevated in the hilus and CA3, but these increases did not reach statistical significance. However, there was no increase in IL-1β levels in the CA1 area. B355252 markedly reduced the IL-1β immunoreactivity in the cerebral cortex, Cpu, and hilus (*p* < 0.01 vs. LPS24h).

Moreover, Western blotting using cortical samples showed that IL-18 moderately increased after 24 h of LPS injection, and B355252 reduced this increase (Figure 9C,D).

### 3.7. B355252 Suppressed the LPS-Elicited Neuroinflammation

**Figure 10:**
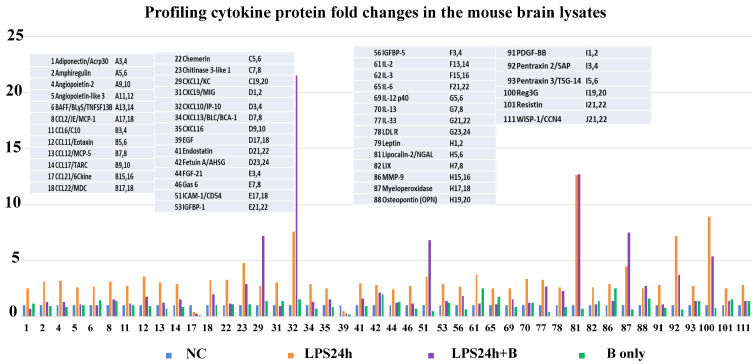
Effect of B355252 on protein expression of cytokines in the cortical samples using a 2.5-fold increase and a 50% decrease as cutoffs. LPS increased the levels of forty-two cytokines and decreased two. B355252 suppressed most of the LPS-induced cytokines.

To assess the impact of B355252 on inflammation, the Proteome Profiler Mouse XL Cytokine Array was utilized to analyze mouse brain lysates. The values of each detected cytokine obtained from control animals were normalized as 1 (blue bars in Figure 10), and the relative changes were calculated by comparing values from other groups (LPS24h, orange bars; LPS24h + B355252, purple bars; and B355252, green bars in Figure 10). Using a cutoff equal to or greater than a 2.0-fold increase and equal to or greater than a 50% decrease, seventy-five cytokines were identified as having increased and two as having decreased. Among them, forty-two showed increases of more than 2.5 fold.

Treatment with B355252 suppressed the majority of LPS-induced cytokine increases, except for four cytokines which further increased (CXCL1/KC, CXCL10/IP-10C, ICAM-1/CD54, and myeloperoxidase), and one remained unchanged (Lipocalin-2/NGAL). Two cytokines, CCL21/6ckine and EGF, were significantly suppressed by LPS at 24 h, and B355252 failed to restore their levels (Figure 10). B355252 alone also increased the levels of two cytokines (IL-2 and MMP-9). The names of these 44 elevated and depressed cytokines are given in Figure 10, while all values of the 111 detected cytokines are provided in Supplemental Table S1.

## 4. Discussion

Our results demonstrate that the small molecular compound B355252 suppresses LPS-elicited neuroinflammatory responses in brain tissues. A single-dose injection of LPS lowered the animal behavioral score and led to cell death in the cortex, Cpu, and hippocampal subregions, as well as increases in Iba-1-positive microglial cells and GFAP-positive astrocytes at 4 h and 24 h of post-LPS injection. Furthermore, LPS increased the immunoreactivities and/or protein levels of inflammatory markers such as TLR4, NLRP3, caspase-1, IL-1β, and IL-18, and induced a 2.5-fold increase in forty-two cytokines. B355252 successfully improved animal behavioral scores, reduced the number of cell deaths, microglial cells, and astrocytes, decreased TLR4, NLRP3, caspase-1, IL-1β, and IL-18, and suppressed the majority cytokines elevated by LPS.

Our results confirm that systemic LPS injection induces neuroinflammatory responses, as represented by activations of microglia and astrocytes and increases in inflammatory cytokines in the brain. Previous studies have demonstrated that LPS disrupts the blood–brain barrier (BBB) [31,32]. Therefore, LPS systemic injection into animals has been broadly used by neuroscience researchers as a neuroinflammation model. While in vitro experiments have established that B355252 potentiates NGF-promoted neurite growth and possesses neuroprotective effects against glutamate-, 6-hydroxydopamin-, and cobalt-induced cell death in cell cultures [1,2,3,4,5,6], to date, only one in vivo study has been conducted, and the results showed that B355252 reduced infarct volume and inhibited neuroinflammation, as evidenced by reductions in ROS accumulation, IL-1β content, and microglia and astrocyte activation [8]. In the present study, we induced an inflammatory model in mice via intraperitoneal LPS injection and examined the anti-inflammatory effects of B355252. LPS significantly reduced animal behavioral scores, with nine out of eleven animals showing declined scores after 24 h of LPS injection, characterized by reduced active body movements and slowed reactions to stimuli. These behavioral changes could either be caused by the inflammatory response in the whole-body systems or damage to the brain, especially to the hippocampus.

LPS resulted in mild neuronal damage in the cortex, Cpu, CA1, and CA3 4 h post-LPS injection. The damage in the hilus region is significantly high. Using Fluoro Jade staining, Ekdahal and colleagues observed pronounced neuronal death in the hippocampal hilus region after LPS injection [33], suggesting that the hilus region may be more vulnerable than other brain regions to LPS-induced injury. The numbers of damaged neurons were further escalated at 24 h post-LPS injection. B355252 significantly ameliorated the above alterations caused by LPS at 24 h, demonstrating that B355252 possesses a neuroprotective effect in an LPS-induced inflammatory model in vivo. It has not been examined whether B355252 can pass through the BBB. However, in one study, Wang and colleagues injected B355252 intraperitoneally in mice subjected to ischemic stroke and their results demonstrated remarkable neuroprotection in reducing ischemic brain damage and suppressing neuroinflammation by B355252 [8]. In another study, our group examined whether B355227, an analog of B355252, could pass through the BBB using an in vitro model of the BBB. The result demonstrated that the BBB is permeable to B355227 [9]. Although there is no direct evidence proving the passage of B355252 through the BBB, these two experiments presented a strong probability.

Our findings indicate that B355252 exhibits an anti-inflammatory effect in the brain. Neuroinflammation, characterized by the activation of microglial cells and astrocytes [34,35,36], was observed following LPS systemic injection. Microglia normally display a ramified morphology with extended or retracted dendrites under physiological conditions. However, in pathological conditions, microglia undergo hyper-ramification, with elongated dendrites and increased branching in the early stages of activation. This progresses to dendrite retraction and thickening, culminating in an amoeboid shape for phagocytosis [37]. Despite not observing Iba-1 positively labeled amoeboid microglial cells even after 24 h of LPS injection, our results suggest that the microglial cells’ activation was in its early or intermediate stages.

Astrocyte activation is typically characterized by an increase in the number of GFAP-positive-stained cells, enlarged soma, and increased dendritic processes [35,38]. These morphological changes were evident in animals injected with LPS after 4 h, most prominently after 24 h, indicating an accelerated neuroinflammatory response. B355252 effectively suppressed the activation of both microglial and astrocytic cells, highlighting its potent anti-inflammatory properties. Considering that a B355252 analog with similar molecular structure can permeate the BBB [9], further exploration of B355252 in vivo application is warranted. We anticipate that our study will broaden the preclinical applications of B355252 in the short term and facilitate its clinical translation as a potentially valuable therapy for mitigating neuroinflammatory responses overall.

Our findings suggest that B355252 may attenuate LPS-induced neuroinflammation and inhibit the pyroptotic pathway. The key steps in pyroptosis activation involve the formation of NLRP3-mediated inflammasome and subsequent caspase-1 activation, IL-1 and IL18 cleavage, and N terminal release from GSDMD. Toll-like receptor 4 (TLR4) plays a crucial role in the immune system by recognizing pathogen-associated molecular patterns (PAMPs). Recent studies have shown that TLR4 exacerbates microglial pyroptosis through NLRP3 inflammasome activation [39,40]. Our results revealed that LPS increased the levels of TLR4, NLRP3, cleaved caspase-1, IL-1β, and IL-18, which is in agreement with previous publications showing activation of TLR4 and NLRP3 by LPS [28,29,41]. B355252 suppressed TLR4, NLRP3, caspase-1, IL-1β, and IL-18 in LPS-injected animals, demonstrating that B355252 is capable of inhibiting the LPS-activated pyroptosis signaling pathway. Pyroptosis has been shown to be involved in the pathogenesis of cardiovascular diseases, metabolic diseases, and neurological diseases (for reviews, see [42,43,44,45,46,47,48]). In the nervous system, pyroptosis is associated with Parkinson’s disease, Alzheimer’s disease, amyotrophic lateral sclerosis (ALS), and cerebral stroke. Therefore, pyroptosis has emerged as a novel therapeutic target for various diseases [48,49,50]. For example, activation of pyroptosis suppresses tumor growth and promotes anti-cancer drug sensitivity [51,52]; while inhibition of pyroptosis prevents neuronal death and improves behavioral performance in Parkinson’s disease, Alzheimer’s disease, ischemic stroke, traumatic brain and spinal cord injuries, and diabetic encephalopathy models [53,54,55,56,57,58,59,60,61].

Furthermore, our results demonstrate that LPS leads to an increase of 75 or 42 cytokines when using 2.0- or 2.5-fold elevation as cutoffs, respectively. These increased cytokines could be classified into six subgroups: interleukins (IL-2, IL-3, IL-6, IL-12 p40, IL-13, IL-33), TNF cytokines (BAFF/TNFRSF13B, Pentraxin 2/SAP, Pentraxin 3/TSG-14), C-C and C-X motif chemokines (CCL2/JE/MCP-1, CCL16/C10, CCL11/Eotaxin, CCL12/MCP-5, CCL17/TRAC, CCL22/MDC, CXCL1/KC, CXCL9/MIG, CXCL10/IP-10, CXCL13/BLC/BCa-1, CXCL16, LIX), growth factor cytokines (Amphiregulin, Angiopoietin 2, Endostatin, FGF21, Gas6, IGFBP-1, IGFBP5, PDGF-BB, Reg3G, WISP-1/CCN4), and metabolism-regulating cytokines (Adiponectin, Angiopoietin-like protein 3, chemerin, chitinase 3-like-1, fetuin, LDLR, leptin, Resistin). Additionally, there were increased levels of neutrophil gelatinase-associated lipocalin (NGAL/LCN2/Lipocalin-2), matrix metalloproteinase 9 (MMP-9), myeloperoxidase (MPO), and osteopontin (OPN). These results demonstrate that LPS can induce broad inflammatory responses in the CNS, and B355252 efficiently suppresses almost all the LPS-triggered cytokines.

The broad-range increases in cytokines could be the consequence of pyroptosis pathway activation or independence. Interleukins, TNF cytokines, and c-c and c-x chemokines could be released as part of an inflammatory response triggered by pyroptotic cell death [62,63,64,65]. Inhibiting TNF-α by CC-5013 has been shown to suppress pyroptosis signaling in the liver and kidneys through a caspase-1 independent pathway [66]. Although the relationships between growth factor cytokines, metabolism-regulating cytokines, and pyroptosis are not well defined, nonetheless, B355252 inhibited the majority of the LPS-triggered cytokine elevation, suggesting the B355252 possesses a broad anti-inflammation effect in the CNS.

Two cytokines, CCL21/6Ckine and EGF, exhibited decreases of more than 50% in LPS-injected animals. CCL21/6Ckine, a highly expressed chemokine in secondary lymphoid organs such as lymph nodes and the spleen, plays a crucial role in adaptive immune responses and inflammation. In the CNS, CCL21 drives CD4^+^ T cell proliferation and migration into the CNS parenchyma [67]. Overexpressing CCL21 induces significant neuroinflammatory responses in the brain [68]. EGF (epidermal growth factor) promotes epidermal keratinocyte proliferation and differentiation and mitigates inflammation [69]. While it is understandable that LPS suppresses the anti-inflammatory EGF, it remains unclear as to why LPS decreases the pro-inflammatory CCL21 as well.

B355252 alone increased the levels of two cytokines: IL-2 and MMP-9. IL-2 (interleukin-2) regulates both pro- and anti-inflammatory responses. On the one hand, it promotes T cell proliferation and differentiation and activates the RAS-ERK signaling pathway; on the other hand, it activates the TORC1 signaling pathway, which has been shown to be associated with energy metabolism and enhanced inflammation [70,71]. MMP-9 regulates extracellular matrix degradation and remodeling. In the CNS, MMP-9 promotes neuroinflammatory processes [72]. The observed increase in IL-2 and MMP-9 suggest that B355252 alone may fine tune the inflammatory processes in the brain.

The present study has the following limitations: (1) although we have employed statistical methods that are robust with respect to smaller sample sizes, one should be cautious when interpreting the results due to the low numbers of animals in each group for the histology and biochemical analyses; (2) a single-dose injection of LPS was used to induce neuroinflammation; in clinic, chronic inflammatory responses may serve as one of the underlying pathogenesis causing chronic neurodegenerative disorders; thus, exploring the effects of repeated low-dose LPS injection in relation to chronic neural degeneration may shed light on the pathogenesis of neurodegenerative disorders; (3) though indirect evidence suggests that B355252 may pass through the BBB, no control of B355252 permeation into the brain tissue was performed.

## 5. Conclusions

In summary, our results confirmed that LPS activated the pyroptotic pathway and caused prominent neuroinflammatory responses. Furthermore, we demonstrated that B355252 possesses a potent anti-inflammatory effect in the brains of animals systemically injected with LPS. This effect may be related to its ability to suppress NLRP3-mediated pyroptotic signaling and pan-cytokine release. The therapeutic effects of B3555252 warrant further examination in other disease models.

## Data Availability

The data that support the findings of this study are available on reasonable request from the corresponding author. The data are not publicly available due to ethical considerations.

## Supplementary Materials

The following supporting information can be downloaded at: https://www.mdpi.com/article/10.3390/brainsci14050467/s1, Figure S1: TUNEL Staining; Table S1: Protein expression of 111 cytokines in each group.
