# Supplementary material for: B355252 Suppresses LPS-Induced Neuroinflammation in the Mouse Brain
Source: Brain Sci. 2024 May 7;14(5):467. doi: 10.3390/brainsci14050467 (PMC11119117; doi:10.3390/brainsci14050467)
Supplement: Supplementary file 1 [file brainsci-14-00467-s001.zip › brainsci-2963305-supplementary.pdf]

Figure S1. TUNEL Staining.

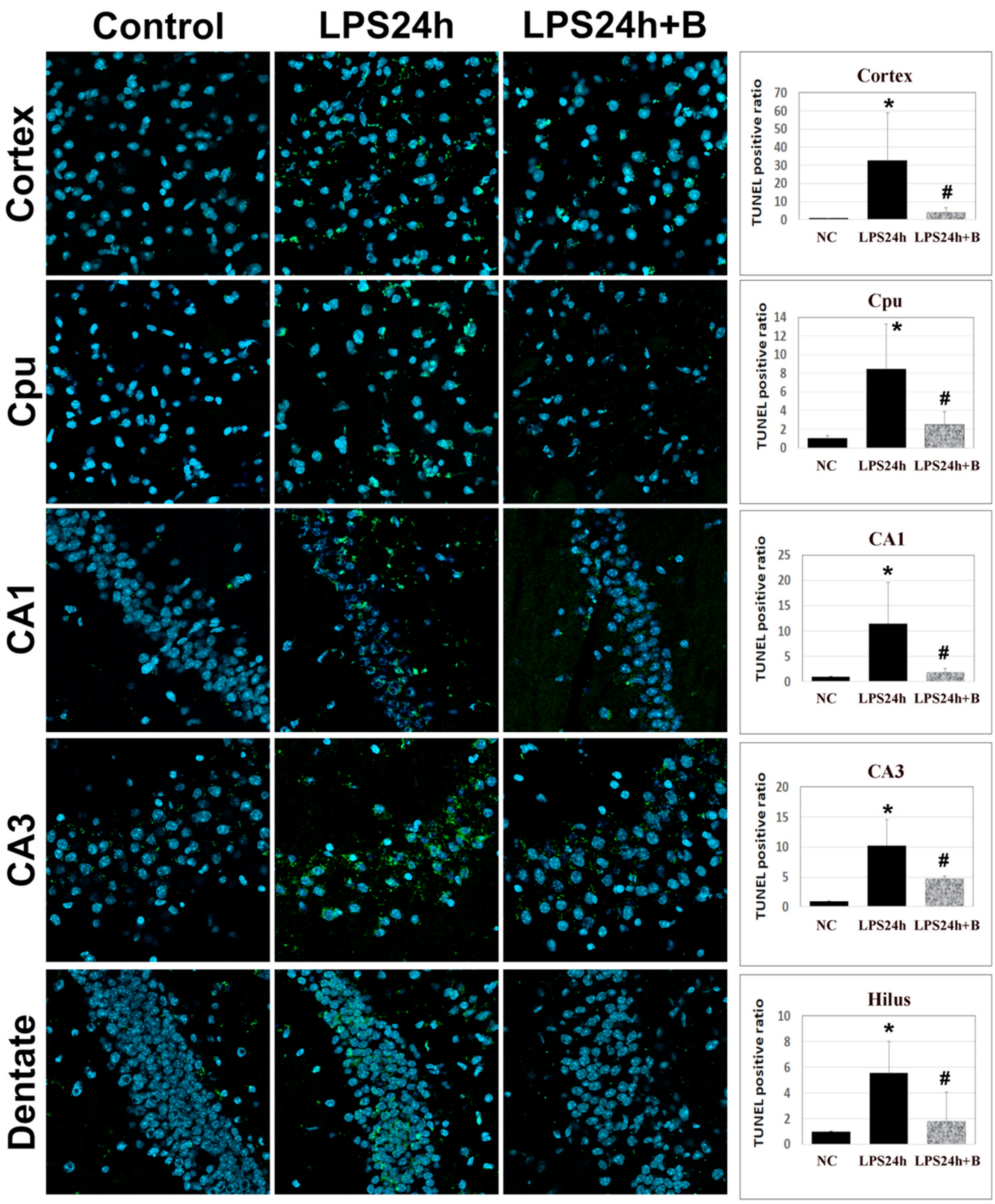

Green color, TUNEL; Blue color, DAPI.

Table S1. Protein expression of 111 cytokines in each group.
Values in NC were set as 1 and relative fold changes were given in LPS24h, LPS24+B and d B groups.

Analyte	Location	Number	NC	LPS24h	LPS24h+B	B only
Adiponectin/Acrp30	A3,4	1	1	2.523	0.732	1.167
Amphiregulin	A5,6	2	1	3.154	1.305	0.907
Angiopoietin-1	A7,8	3	1	2.242	1.298	0.927
Angiopoietin-2	A9,10	4	1	3.157	1.335	0.822
Angiopoietin-like 3	A11,12	5	1	2.620	1.065	0.972
BAFF/BLyS/TNFSF13B	A13,14	6	1	2.674	1.044	1.493
C1q R1/CD93	A15,16	7	1	2.441	1.026	0.736
CCL2/JE/MCP-1	A17,18	8	1	3.152	1.502	1.396
CCL3/CCL4/MIP-1α/β	A19,20	9	1	2.253	1.392	1.373
CCL5/RANTES	A21,22	10	1	2.331	6.271	0.820
CCL6/C10	B3,4	11	1	2.763	1.134	1.022
CCL11/Eotaxin	B5,6	12	1	3.582	1.772	0.918
CCL12/MCP-5	B7,8	13	1	3.050	1.219	0.722
CCL17/TARC	B9,10	14	1	2.913	1.573	0.886
CCL19/MIP-3β	B11,12	15	1	2.073	1.324	0.914
CCL20/MIP-3α	B13,14	16	1	1.920	0.979	1.460
CCL21/6Ckine	B15,16	17	1	0.377	0.230	0.122
CCL22/MDC	B17,18	18	1	3.279	1.985	0.978
CD14	B19,20	19	1	1.982	1.373	0.666
CD40/TNFRSF5	B21,22	20	1	2.283	1.944	1.041
CD160	C3,4	21	1	2.094	1.175	0.873
Chemerin	C5,6	22	1	3.276	1.187	1.109
Chitinase 3-like 1	C7,8	23	1	4.807	2.907	1.115
Coagulation Factor III/Tissue Factor	C9,10	24	1	1.121	1.205	1.204
Complement Component C5/C5a	C11,12	25	1	1.743	0.876	0.884
Complement Factor D	C13,14	26	1	1.533	0.776	1.122
C-Reactive Protein/CRP	C15,16	27	1	1.609	0.722	0.447
CX3CL1/Fractalkine	C17,18	28	1	0.778	0.969	0.860
CXCL1/KC	C19,20	29	1	2.755	7.182	1.349
CXCL2/MIP-2	C21,22	30	1	2.413	1.994	0.843
CXCL9/MIG	D1,2	31	1	3.012	0.925	1.378
CXCL10/IP-10	D3,4	32	1	7.566	21.554	1.567
CXCL11/I-TAC	D5,6	33	1	2.272	1.260	1.359
CXCL13/BLC/BCA-1	D7,8	34	1	2.878	1.288	0.676

CXCL16	D9,10	35	1	2.495	1.564	0.841
Cystatin C	D11,12	36	1	1.489	1.900	2.292
DKK-1	D13,14	37	1	1.371	1.025	1.346
DPPIV/CD26	D15,16	38	1	1.167	0.943	0.794
EGF	D17,18	39	1	0.449	0.235	0.186
Endoglin/CD105	D19,20	40	1	1.189	0.964	0.498
Endostatin	D21,22	41	1	3.000	1.599	0.949
Fetuin A/AHSG	D23,24	42	1	2.810	2.165	1.992
FGF acidic	E1,2	43	1	1.526	1.748	2.055
FGF-21	E3,4	44	1	2.466	1.255	1.277
Flt-3 Ligand	E5,6	45	1	2.041	1.060	0.619
Gas 6	E7,8	46	1	2.780	1.175	0.704
G-CSF	E9,10	47	1	2.050	1.666	1.208
GDF-15	E11,12	48	1	2.097	1.008	0.594
GM-CSF	E13,14	49	1	2.095	2.320	1.308
HGF	E15,16	50	1	1.379	0.765	0.480
ICAM-1/CD54	E17,18	51	1	3.584	6.811	0.488
IFN-γ	E19,20	52	1	2.364	1.139	1.289
IGFBP-1	E21,22	53	1	2.871	1.421	1.259
IGFBP-2	E23,24	54	1	1.540	1.064	0.833
IGFBP-3	F1,2	55	1	2.351	1.248	0.846
IGFBP-5	F3,4	56	1	2.686	1.852	0.665
IGFBP-6	F5,6	57	1	1.637	1.434	0.758
IL-1α/IL-1F1	F7,8	58	1	2.337	1.080	1.234
IL-1β/IL-1F2	F9,10	59	1	1.840	1.379	1.413
IL-1ra/IL-1F3	F11,12	60	1	1.723	1.180	1.190
IL-2	F13,14	61	1	3.740	1.174	2.505
IL-3	F15,16	62	1	2.309	1.582	1.976
IL-4	F17,18	63	1	1.654	1.390	1.615
IL-5	F19,20	64	1	1.911	0.965	1.420
IL-6	F21,22	65	1	2.521	1.093	1.759
IL-7	F23,24	66	1	2.253	1.347	0.668
IL-10	G1,2	67	1	2.106	1.229	1.219
IL-11	G3,4	68	1	2.431	1.469	1.210
IL-12 p40	G5,6	69	1	2.490	1.505	0.863
IL-13	G7,8	70	1	3.355	1.246	1.272
IL-15	G9,10	71	1	1.417	1.493	1.169
IL-17A	G11,12	72	1	1.772	1.215	2.066
IL-22	G13,14	73	1	1.873	0.955	1.803

IL-23	G15,16	74	1	1.985	0.850	1.632
IL-27 p28	G17,18	75	1	2.084	1.422	1.307
IL-28A/B	G19,20	76	1	2.035	1.166	0.931
IL-33	G21,22	77	1	3.238	2.683	0.435
LDL R	G23,24	78	1	2.565	2.328	0.836
Leptin	H1,2	79	1	2.366	1.563	1.646
LIF	H3,4	80	1	1.580	1.239	1.061
Lipocalin-2/NGAL	H5,6	81	1	12.597	12.702	0.697
LIX	H7,8	82	1	2.588	1.098	1.414
M-CSF	H9,10	83	1	1.577	1.601	1.173
MMP-2	H11,12	84	1	1.631	0.743	0.619
MMP-3	H13,14	85	1	2.423	1.051	1.956
MMP-9	H15,16	86	1	2.871	1.412	2.507
Myeloperoxidase	H17,18	87	1	4.501	7.489	0.613
Osteopontin (OPN)	H19,20	88	1	2.506	2.765	1.615
Osteoprotegerin/TNFRSF11B	H21,22	89	1	2.121	1.102	1.512
PD-ECGF/Thymidine phosphorylase	H23,24	90	1	1.973	0.930	1.187
PDGF-BB	I1,2	91	1	2.854	1.112	0.762
Pentraxin 2/SAP	I3,4	92	1	7.200	3.700	0.647
Pentraxin 3/TSG-14	I5,6	93	1	2.768	1.349	1.381
Periostin/OSF-2	I7,8	94	1	1.647	1.176	2.008
Pref-1/DLK-1/FA1	I9,10	95	1	0.953	1.188	0.850
Proliferin	I11,12	96	1	2.317	1.234	1.352
Proprotein Convertase 9/PCSK9	I13,14	97	1	2.101	1.130	0.619
RAGE	I15,16	98	1	1.398	0.693	0.526
RBP4	I17,18	99	1	1.495	1.482	0.935
Reg3G	I19,20	100	1	8.960	5.390	0.797
Resistin	I21,22	101	1	2.534	1.383	1.558
E-Selectin/CD62E	J3,4	102	1	2.163	1.349	1.242
P-Selectin/CD62P	J5,6	103	1	2.438	1.192	1.979
Serpin E1/PAI-1	J7,8	104	1	2.171	0.989	1.537
Serpin F1/PEDF	J9,10	105	1	0.946	0.717	1.107
Thrombopoietin	J11,12	106	1	2.115	1.151	1.510
TIM-1/KIM-1/HAVCR	J13,14	107	1	2.422	1.478	2.247
TNF-α	J15,16	108	1	2.025	1.127	1.129
VCAM-1/CD106	J17,18	109	1	1.696	1.745	0.757
VEGF	J19,20	110	1	1.846	1.103	1.179
WISP-1/CCN4	J21,22	111	1	2.824	1.352	1.421
